# Broad-Spectrum Inhibition of HIV-1 by a Monoclonal Antibody Directed against a gp120-Induced Epitope of CD4

**DOI:** 10.1371/journal.pone.0022081

**Published:** 2011-07-19

**Authors:** Samuele E. Burastero, Barbara Frigerio, Lucia Lopalco, Francesca Sironi, Daniela Breda, Renato Longhi, Gabriella Scarlatti, Silvana Canevari, Mariangela Figini, Paolo Lusso

**Affiliations:** 1 Department of Immunology, Transplantation and Infectious Diseases, San Raffaele Scientific Institute, Milan, Italy; 2 Unit of Molecular Therapies, Department of Experimental Oncology and Laboratories Fondazione IRCCS, National Institute of Tumors, Milan, Italy; 3 Institute of Chemistry of Molecular Recognition, National Research Council, Milan, Italy; Mayo Clinic, United States of America

## Abstract

To penetrate susceptible cells, HIV-1 sequentially interacts with two highly conserved cellular receptors, CD4 and a chemokine receptor like CCR5 or CXCR4. Monoclonal antibodies (MAbs) directed against such receptors are currently under clinical investigation as potential preventive or therapeutic agents. We immunized Balb/c mice with molecular complexes of the native, trimeric HIV-1 envelope (Env) bound to a soluble form of the human CD4 receptor. Sera from immunized mice were found to contain gp120-CD4 complex-enhanced antibodies and showed broad-spectrum HIV-1-inhibitory activity. A proportion of MAbs derived from these mice preferentially recognized complex-enhanced epitopes. In particular, a CD4-specific MAb designated DB81 (IgG1Κ) was found to preferentially bind to a complex-enhanced epitope on the D2 domain of human CD4. MAb DB81 also recognized chimpanzee CD4, but not baboon or macaque CD4, which exhibit sequence divergence in the D2 domain. Functionally, MAb DB81 displayed broad HIV-1-inhibitory activity, but it did not exert suppressive effects on T-cell activation *in vitro*. The variable regions of the heavy and light chains of MAb DB81 were sequenced. Due to its broad-spectrum anti-HIV-1 activity and lack of immunosuppressive effects, a humanized derivative of MAb DB81 could provide a useful complement to current preventive or therapeutic strategies against HIV-1.

## Introduction

The CD4 protein expressed on the membrane of T lymphocytes and mononuclear phagocytic cells serves as the primary HIV-1 receptor, which mediates binding of the viral envelope (Env) to the cellular membrane. Additional receptor molecules such as the chemokine receptors CCR5 and CXCR4 function as obligatory coreceptors for HIV-1 entry [1], [2], [3]. Binding of Env to CD4 initiates a chain of events, including major conformational changes in the external Env glycoprotein gp120 that reshuffle its fine antigenic structure[4]. This process leads to the unraveling or *de novo* formation of specific epitopes, which are critically involved in subsequent interactions with the coreceptors [5], culminating in the exposure of the hydrophobic fusion domain of the transmembrane envelope subunit, gp41. Fusion of the apposed cellular and viral membranes ensues [5]. Antibodies that block HIV-1 Env-mediated fusion typically interfere with the binding of CD4 with gp120, but several neutralizing antibodies that interfere with post-binding events have also been described [5], [6], [7], [8]. In particular, antibodies directed towards determinants positioned far from the receptor-binding site have been identified in sera from gp120-immunized animals [9], [10], in patient sera with strong neutralizing activity, and in antibody libraries obtained from HIV-1-seropositive individuals [6], [11], [12]. This property is not exclusive to HIV-1, as it was also reported for antibodies elicited by herpes simplex virus and Epstein-Barr virus [13], [14].

Besides Env-specific antibodies, CD4-targeted antibodies may also be involved in HIV-1 inhibition both at the binding and post-binding levels. We previously identified anti-CD4 antibodies in both European [15] and Asian [16] HIV-1-seronegative individuals who were apparently protected from infection despite repeated exposure to HIV-1 through an infected sexual partner. These antibodies included binding to epitopes exposed on the receptor-Env complex that were correlated with *in vitro* inhibition of HIV-1-induced cell fusion [16]. In all of these circumstances, it appears that antibodies that recognize determinants that participate in post-binding steps can interrupt the chain of events leading to HIV-1 entry into the cell.

Despite worldwide efforts, attempts to develop a protective anti-HIV vaccine have been thus far unsuccessful [17]. Several reasons may underlie this failure, including the elusive antigenic make up of the HIV-1 Env, which is extremely efficient in escaping immunologic control, and the need to achieve *bona fide* sterilizing immunity in the case of a chromosomally-integrating retrovirus, which is beyond the reach of conventional vaccines [18]. A promising strategy for the induction of broadly reactive antibodies is based on the use of immunogens presenting non-polymorphic epitopes that are expressed on the HIV-1 entry complex, i.e., the Env-receptor complex. Immunization with a single-chain chimeric molecule encompassing HIV-1 gp120 bound to a truncated form of human CD4 has yielded some degree of protection in a macaque model [19]. It is worth noting that the focus in these attempts was restricted to epitopes expressed on the HIV-1 component. However, it has been shown that invariant epitopes expressed on the receptor and coreceptor may also be effectively targeted by neutralizing antibodies. Indeed, a non-immunosuppressive anti-CD4 monoclonal antibody (MAb) that does not interfere with gp120 binding [20] and a CCR5-specific MAb (PRO-140) [21] are currently under clinical investigation as potential therapeutic or preventive treatments. The non-polymorphic nature of these cellular antigens makes these approaches worth of further investigation also in the framework of active immunization protocols.

In this study, we used a novel immunization approach based on fusion-competent native Env-CD4 molecular complexes in a mouse model with the aim of eliciting broadly reactive neutralizing antibodies. We describe herein the specificity and function of a MAb, designated DB81, that recognizes a complex-enhanced epitope on human CD4. This MAb inhibits cell fusion and viral replication by divergent HIV-1 strains by a post-binding mechanism and exerts little, if any, suppressive effects on T-cell activation *in vitro*, thereby providing a potentially useful complement to current anti-HIV-1 strategies.

## Materials and Methods

### Cells

NIH 3T3, HeLa, Sup-T1 and RK13 cells were obtained from the American Type Culture Collection. The PM1 cell clone has been described elsewhere [22] and is available from the AIDS Research and Reference Reagent Program (Rockville, MD). All cell lines were maintained at 37°C in a 5% CO2 atmosphere and grown in RPMI 1640 medium (Gibco, Invitrogen Life Technologies, Milan, Italy) supplemented with 10% heat-inactivated fetal bovine serum (FBS, Hyclone, Logan, UT, USA), 2 mM L-glutamine and antibiotics (Gibco); this medium will be hereafter referred to as complete medium. The 8- azaguanine resistant, non-secreting P3X63Ag8.653 mouse myeloma (American Type Culture Collection, Manassas, VA, USA) was maintained in RPMI 1640 medium with 2 mM L-glutamine adjusted to contain 1.5 g/L sodium bicarbonate, 4.5 g/L glucose, 10 mM HEPES, and 1.0 mM sodium pyruvate, 90%; fetal bovine serum, 10%. P3X63Ag8.653 was used as fusion partner for producing hybridomas.

### Expression of Env by recombinant vaccinia vectors

Vaccinia virus was used as a vector to express HIV-1 Ba-L Env on NIH 3T3 effector cells [23]. The vCB-43, HIV-1 Ba-L Env [24], derived from the WR parental strain, was used for this purpose. Target cells used for the functional evaluation of fusion competence of soluble CD4-activated immunogens [25] were induced to express the CCR5 coreceptor using plasmid pGA9-CKR5 [26]. Env-mediated cell fusion was quantified with a previously described vaccinia virus-based reporter gene assay [27].

### Proteins, peptides and antibodies

The following recombinant proteins were provided by NIH AIDS Research and Reagent program, Rockville, (MD): two-domain (2D) soluble CD4 (amino acids 1 to 183, produced in *E. coli*); 4-domain (4D) soluble CD4 (amino acids 1 to 369, produced in CHO cells), human MAb 17b, recombinant HIV-1 IIIB gp120 and recombinant HIV-1 Ba-L gp120. RANTES (Regulated on Activation, Normal T-cell Expressed and Secreted) was purchased from R&D Systems (Minneapolis, MN, USA). The Leu3a mouse MAb anti-human CD4, specific to the gp120-binding site, was purchased from Becton-Dickinson Biosciences (Milan, Italy). Rabbit ATG (thymoglobulin) was obtained from Imtix Sangstat (Milan, Italy). Rabbit complement was obtained from One Lambda Inc. (Canoga park CA,USA). Phytoemoagglutinin (PHA) was from Sigma Italia (Milan, Italy) and tetanus toxoid from Connaught Laboratories Inc. (Toronto, Canada). The Full-Length Single Chain (FLSC) construct consisting of full-length HIV-1Ba-L gp120 and the D1D2 domains of CD4 joined by a 20-amino-acid linker [28] was kindly provided by Dr. A. De Vico. Two 19-mer syntethic peptides (GTWTCTVLQNQKKVEFKID and GTWTCTVSQDQKTVEFKID) were synthesized by solid-phase F-moc using an Applied Biosystems 433A peptide synthesizer (Foster City, CA) and were purified by semi-preparative Reverse Phase-High Performance Liquid Chromatography (RP-HPLC). Linear gradients of acetonitrile in water/0.1% trifluoroacetic acid were used to elute the bound peptides. The purity of the peptides (over 95%) was confirmed by analytical RP-HPLC, and their mass was determined by matrix-assisted laser desorption/ionization time-of-flight analysis with a Voyager-RP Biospectrometry Workstation (PE Biosystem, Inc.). Observed experimental mass values were in agreement with the theoretical calculated ones. The 27-amino acid CD4M3 miniprotein, which was designed on the basis of structural similarity between the CDR2-like loop of the first CD4 domain and the beta-hairpin region of scyllatoxin[29] was a generous gift from the late C. Vita.

### Preparation and validation of the immunogens

NIH 3T3 cells were infected with gp160-expressing [24] recombinant vaccinia viruses overnight at 32°C to induce expression of the recombinant proteins. The following day, if appropriate, the cells were treated with 2D-CD4 (20 µg per million cells) for 30 min. on ice and subsequently washed twice with PBS to remove unbound soluble CD4. The cells used for immunization were fixed with paraformaldehyde for 10 min at room temperature at a cell density of 1×10^6^ cells per ml, washed twice in PBS and checked by flow cytometry for the expression of a CD4-induced epitope, 17b, before injection. The 17b MAb is directed to the coreceptor-binding domain of gp120 and only faintly reacts with Env-expressing cells in the absence of soluble CD4 [30]. Only the preparations of immunogens displaying a CD4-dependent increase of 17b binding to Ba-L gp120 expressed by vCB43 infected NIH 3T3 cells after fixation were considered suitable as immunogens for inoculation. A functional quality control was also performed, aimed at verifying the competence for fusion of CD4-activated immunogens. In this case, the effector cell population were infected with vaccinia virus expressing bacteriophage T7 RNA polymerase encoded by the vP11T7gene 1 [31], and the target cell population with a vaccinia virus expressing the *lacZ* reporter gene under the control of the T7 promoter (plasmid pG1NT7-gal, R. A. Morgan, National Human Genome Research Institute). Additionally, target cells were transfected with a plasmid expressing the CCR5 coreceptor (GA9-CKR5) [26] or the CXCR4 coreceptor as a control (pYF1-fusin) [32]. Effector and target cells were mixed in duplicate wells of 96-well plates (2×10^5^ cells of each type per well). As negative controls, the cells were incubated with buffer alone. Plates were incubated for 2.5 h at 37°C, and fusion was quantified by measurement of beta-galactosidase activity in nonionic detergent cell lysates, using a 96-well spectrophotometer (Titertek, Huntsville, Alabama, USA).

### Animals and immunization protocol

Four to 5 weeks old female Balb/c mice bred by Charles River (Calco, Italy) were used for immunizations, which were performed in a standard-pathogen-free animal facility. Four mice per each experimental condition were used. Subcutaneous injections were made in the dorsal part of the neck. A total of 5×10^5^ cells in 250 µl of PBS were used per inoculation. A total of 4 inoculations were made at 20- to 25-day intervals, and blood was drawn from the tail vein 10–14 days after the last recall injection. All animal work have been conducted according to national and international guidelines. In particular, animals were maintained in standard pathogen free conditions, and every care was taken to minimize the suffering related to experimental work and to ameliorate their living conditions. The study was approved by the Institutional Ethic on Animal Welfare (IACUC) of the San Raffaele Scientific Institute. Namely, the following approvals were obtained, following formal applications: record number 397, July 7th, 2009; record number 222, November 17, 2003; record number 141, March 29, 2001.

### Immunochemistry

ELISA reagents were obtained from Sigma (Milan, Italy), unless differently specified. Binding activity to solid-phase antigens was measured as follows: recombinant molecules were used for coating ELISA plates (Costar, Milan, Italy), diluted in PBS and incubated at 4°C overnight. Recombinant 2D-CD4 (molecular weight: 20,14) was used at 2 µg/ml for coating. CD4-gp120 complexes were formed by pre-incubation of equimolar amounts of the two moieties in ice for 20 min. All subsequent incubations, except for developing, were for 1 h at 20°C; PBS containing 0,05% Tween 20 was used for washing.

Aliquotes of phosphatase-conjugated gp120 were prepared by Lightning Link Alkaline Phosphatase Conjugation Kit Protocol, according to Manufacturer instructions (Novus Biologicals, Cambridge, UK). Blocking of ELISA wells was done with 1% bovine serum albumin (BSA, Sigma) in PBS. Supernatants of hybridomas were tested in triplicate at different dilutions in PBS containing 0.05% Tween 20. Purified antibodies were tested over the indicated dilution ranges. Binding of IgG antibodies was revealed with an affinity-purified, phosphatase-conjugated polyclonal antibody to mouse IgG (Southern Biotech, Birmingham, AL, USA). The reaction was developed with PNPP-developing reagent and read with an automated plate reader by using a 405 nm filter (Titertek, Pharmacia, Uppsala, Sweden). To test inhibition of gp120 binding to solid-phase CD4, after coating the plates with 5 µg/ml rCD4, 50 µl of phosphatase-labeled gp120 (5 µg/ml) was added and incubated for 1 h, plus or minus serial twofold dilutions of the test antiserum. The phosphatase activity was then measured by the rate of PNPP hydrolysis. The inhibition caused by the test antibody was expressed as percent decrease from the binding of untreated control gp120.

### Production of monoclonal antibodies

A conventional protocol for the generation of MAbs from cell hybrids between spleen cells of immunized animals and the P3X63Ag8.653 non-secreting mouse myeloma was used [33]. Antibody purification from cell supernatants was performed by affinity gel with protein-G-coupled sepharose (Pharmacia) according to manufacturer's instructions. Absorption of MAb DB81 (20 nM in 150 µl of PBS) on solid-phase antigens was done on wells coated with antigens and blocked with BSA under the same conditions used in the ELISA assays; 200 microliters of 20 nM MAb solution were transferred from one well to the following in ten sequential steps, with 2 min incubation at 20°C before each step. Uncoated, BSA-blocked wells were used in parallel as controls.

### Flow cytometry

Cells used for flow cytometric analysis (500,000 cells per sample) were incubated for 20 min. on ice with 1 µg of the appropriate MAb, and binding was revealed with a secondary fluorescein-conjugated, affinity-purified polyclonal antibody to mouse IgG used at 0.2 µg per sample (Sigma). A FACScan cytometer equipped with a CellQuest software (Becton Dickinson) was used for acquiring and analyzing the stained samples.

### Viruses and preparation of chronically infected cell lines

The laboratory-passaged isolates IIIB and Ba-L were obtained from the NIH AIDS Research and Reference Reagent Program, NIAID, NIH (Rockville, MD). The HIV-1 primary isolate B117 was kindly provided by Eva M. Fenyo (Lund University, Sweden), whereas the 6195 isolate was obtained from the NIBSC-MRC AIDS Reagent Project (London, UK) within the framework of the WHO-UNAIDS Network for the Characterization of GloBa-Lly Prevalent HIV-1 Strains in Relation to Vaccine Development. All HIV-1 isolates were expanded and titrated in activated primary human peripheral blood mononuclear cells (PBMC), as previously reported [34]. Primary isolates were minimally passaged *in vitro*. The PM1 cell clone and its persistent infection with biologically diverse HIV-1 strains have been described elsewhere [22]. Chronically infected PM1 cell lines were obtained for the B117 and the 6195 HIV-1 isolates, whereas for IIIB and MN isolates, chronically infected SupT1 cell lines were derived. To produce the chronically infected cell lines, cells were exposed to the viral stocks at the approximate multiplicity of infection of 0.1, and subsequently cultured in complete culture medium and monitored daily for cytopathic effects and extracellular p24 antigen release by ELISA using commercial antibodies (Aalto Bio Reagents, Dublin, Ireland). At the peak of the cytopathic effects (typically, day 7-10 post-infection), cells were pelleted, washed once in pre-warmed complete medium and cultured at low cellular density (5 x 104 cells/ml) with daily replacement of half of the culture medium in the presence of conditioned supernatants (20% vol/vol) from uninfected PM1 (or SupT1). In most cases, this treatment resulted in the appearance, within 10 to 14 days, of small clusters of healthy-appearing cells that rapidly re-colonized the cultures. These outgrowing cell lines were chronically infected, as assessed by the stable expression of cell surface viral envelope and by the sustained release of extra-cellular p24 antigen.

### HIV-1 envelope-mediated fusion assay

An HIV-1 Env-mediated fusion assay was used to evaluate HIV-1 inhibition. The assay was performed using a modification of the test, based on vaccinia technology, which was originally developed by E. Berger and coworkers [27]. In the modified assay, the effector cells were chronically infected PM1 or SupT1 cells, whereas the target cells were NIH 3T3 mouse fibroblast cells permanently expressing human CCR5 or CXCR4, along with human CD4. Twelve hours before the test, effector cells were infected with a vaccinia vector expressing bacteriophage T7 RNA polymerase encoded by vP11T7gene 1 [31], while target cells were infected with a vaccinia vector expressing the *lacZ* reporter gene under the control of the T7 promoter, as described (plasmid pG1NT7-gal, R. A. Morgan, National Human Genome Research Institute, personal communication). All vaccinia virus infection were performed in DMEM medium supplemented with 2.5% FBS. The cells were then washed with DMEM 2.5% and the effector cells were mixed for 2 hrs with the target cells in the presence or absence of the inhibitors.

### T-cell proliferation assays

PBMC were isolated from heparin-treated venous blood drawn from tetanus toxoid vaccinated healthy lab workers according to standard protocols based on gradient density (Ficoll Hypaque, Pharmacia, Uppsala, Sweden). Cells were incubated in 96-well, flat-bottom plates (PBI International, Milan, Italy) at 37°C in 5% CO2 atmosphere, at a concentration of one million cells per ml in 200 µl in complete medium. Tetanus toxoid (5 µg/ml) (Connaught Laboratories, Inc, Toronto, Canada) or phytoemoagglutinin (PHA) (10 µg/ml) were added at time 0. Where appropriated, Leu 3a or DB81 MAb (5 µg/ml) was also added from the beginning of the culture. As negative controls, microcultures with medium only were included. 3H-TdR thymidine (DuPont-NEN, Boston, MA) was added (1 µCi per well) in the last 18 hrs of a 3- or 5-days culture time (for PHA- and tetanus toxoid-pulsed cells, respectively). As read-out of proliferation, cells were harvested (Skatron Instruments, Lier, Norway) and thymidine incorporation was measured in a beta-counter (Perkin- Elmer, Shelton, CT, USA).

### Complement fixation

PBMC from healthy lab workers (100,000 per test, in RPMI medium) were reacted (30 min, 37°C) with freshly reconstituted rabbit complement (Harlan Sera Lab, Loughborough, England), according to manufacturer's instruction. As positive control, an incubation (30 min, 37°C) with anti-human thymoglobuline (ATG, Imtix-Sangstat s.r.l., samples 20 nM DB81 or Leu 3a MAb were added, respectively (30 min, 37°C) before complement addition. Washing steps with PBS were included between incubations, as appropriated. The proportion of necrotic cells was evaluated by flow cytometry as propidium iodide staining.

### Surface plasmon resonance

Binding experiments were performed using a Biacore 2000 equipped with research-grade CM5 sensor chips (Biacore AB, Uppsala, Sweden). A standard amine-coupling protocol, with N-hydroxysuccinimide (NHS), 1-Ethyl-3-(3-dimethylaminopropyl)-carbodiimide (EDC) and Ethanolamine hydrochloride (pH 8.5) was used to immobilize the antibody. The immobilization was carried out at a concentration of 40 ng/µl using 10 mM Hepes Buffer pH 7.4, 150 mM NaCl (HSP) containing 0.005% P-20 surfactant (HBS-P) as the running buffer. Flow cell 1 was used as a reference cell. 2D-CD4 previously incubated with gp120 IIIB protein for 1h RT was injected at a flow rate of 30 µl/min for 3 minutes. Sample dilutions were prepared in HBS buffer and binding analyses were performed at a concentration of 200 nM. Kinetics analyses were performed at concentrations ranging from 6.25 to 200 nM.

### Cloning of the MAb DB81 gene

The V genes of mouse MAb DB81 were reverse transcribed and amplified using PCR essentially as described [35]. The variable domains of the antibody were sequenced. Sequencing reactions were performed on amplification products using the ABI PRISM Big Dye Terminator Cycle Sequencing kit v1.1 (Applied Biosystems) and examined on an ABI PRSM 3100 Genetic Analyzer (Applied Biosystems), using the DNA Sequencing Analysis software 3.7 (Applied Biosystems). The following primers were used for the amplification and sequence: V-Light: 5′ primer GACATTGTGATGACCCAGTTTGC; 3′ primer TTTGATTTCCAGCTTGGTGCC V-Heavy: 5′ primer GARGTCCAGCTGCAACAGTCYGGAC; 3′ primer TGCAGAGACAGTGACCAGAG.

### Statistical analysis

Descriptive statistics were used to describe quantitatively the distribution of collected data. Results are shown as mean values ± standard error of the mean.

### Sequence accession numbers

GenBank CAQ16367.1

Anti-human CD4-HIV-1-gp120 complex DB81 monoclonal antibody immunoglobulin heavy chain [Mus musculus]

GenBank CAQ16368.1

Anti-human CD4-HIV-1-gp120 complex DB81 monoclonal antibody immunoglobulin kappa chain [Mus musculus]

## Results

### NIH-3T3 cells expressing soluble CD4-complexed HIV-1 Env elicit an antibody response to complex-specific epitopes

**Figure 1:**
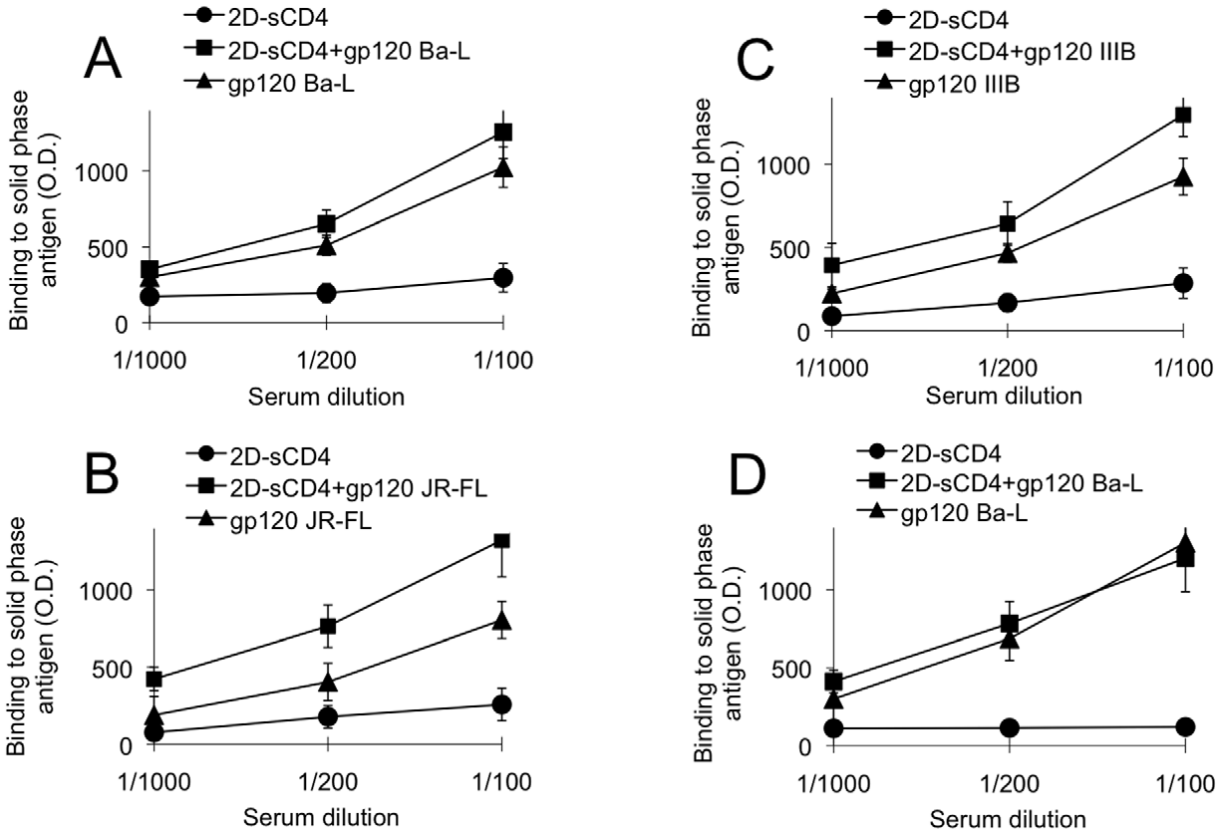
Detection of IgG anti-CD4 and anti-CD4-gp120 complexes in sera from mice immunized with CD4-complexed HIV-1 Env. he binding activity of IgG present in pooled sera obtained from mice immunized with NIH-3T3 cells expressing HIV-1 Env from various viral isolates bound to soluble CD4 (**A,B,C**) or from control mice immunized with cells expressing unbound HIV-1 Env (**D**) was tested by ELISA. The solid phase antigens used were recombinant HIV-1 gp120 complexed with equimolar concentrations of 2D-CD4, recombinant gp120 alone or 2D-CD4 alone. The binding activity was measured not only to Ba-L gp120 (1A and 1D), which is homologous to that used for immunization, but also to gp120 derived from heterologous viral isolates, JR-FL (Figure 1B) and IIIB (1C).

Balb/c mice were immunized with autologous NIH-3T3 cells infected for 24 hours with a recombinant vaccinia virus (vCB-43) encoding the complete HIV-1 Env gp160 gene cloned from an R5 viral isolate (Ba-L), treated with 2D-CD4 (20 µg/10^6^ cells) for 30 minutes and fixed with paraformaldehyde before injection. Control mice were immunized with cells infected with vCB43 but not treated with soluble CD4. Sera obtained from immunized mice were screened for IgG binding activities to solid-phase CD4, gp120 or equimolar CD4-gp120 complexes by ELISA. In all cases, animals immunized with Env/CD4 complexes developed higher IgG binding activities to the CD4-gp120 complex than to either CD4 or gp120 tested separately (Fig. 1A). Of note, this increased binding activity to the CD4-gp120 complex was not dependent on the HIV-1 strain from which gp120 was derived, as similar data were obtained with complexes formed with HIV-1 Ba-L (R5), HIV-1 IIIB (X4) and HIV-1 JRFL (R5) gp120 (Fig. 1A, 1B and 1C). In sera from control mice injected with NIH-3T3 expressing uncomplexed Env (*i.e.*, without addition of 2D-CD4), the IgG binding activities to gp120 and CD4-gp120 complexes were comparable (Fig. 1D).

### HIV-1-neutralizing activity and fine specificity of sera from immunized mice

**Figure 2:**
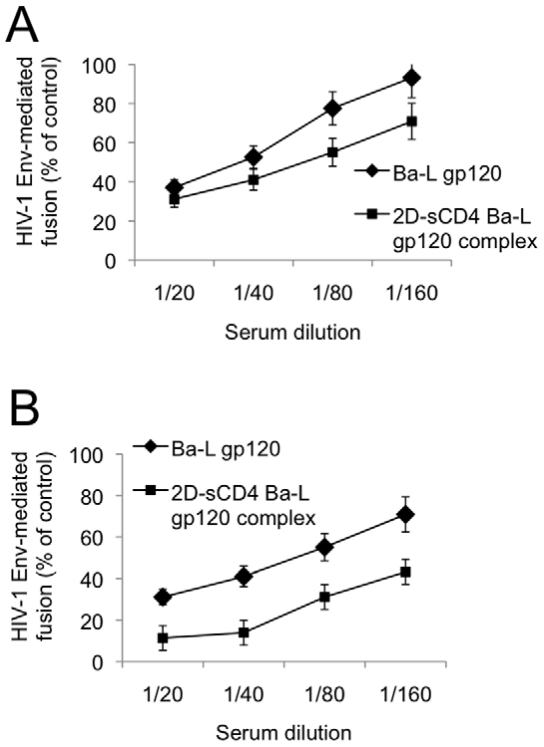
Inhibition of HIV-1 Env-mediated fusion by sera from mice immunized with CD4-complexed HIV-1 Env. era from mice immunized with autologous NIH 3T3 cells expressing HIV-1 Ba-L Env in complex or not with soluble CD4 were tested for inhibition of HIV-1 Env-mediated fusion using PM-1 (**A**) of Sup-T1 (**B**) cells chronically infected with HIV-1 Ba-L or IIIB, respectively, as effector cells. NIH 3T3 cells expressing human CD4 and the appropriate coreceptor (CXCR4 or CCR5) were used as target cells. Sera from 4 immunized mice were pooled. Fusion inhibition is expressed as percent of the positive control, i.e., the amount of fusion measured when the fusion partners were reacted in the presence of pre-immune sera.

Pooled sera derived from mice injected with autologous NIH-3T3 cells expressing the HIV-1 Ba-L Env in complex or not with 2D-CD4 were tested for inhibition of HIV-1 Env-mediated fusion using PM-1 of Sup-T1 cells chronically infected with HIV-1 Ba-L or IIIB, respectively, as effector cells. As shown in Figure 2, sera obtained from mice immunized with sCD4-complexed Env displayed in both cases a stronger fusion-inhibitory activity compared to those obtained from mice immunized with uncomplexed Env. The spectrum of binding and neutralizing activity of sera derived from mice immunized with sCD4-complexed Env will have to be established with systematic studies on a wide range of genetically diverse HIV-1 strains.

**Figure 3:**
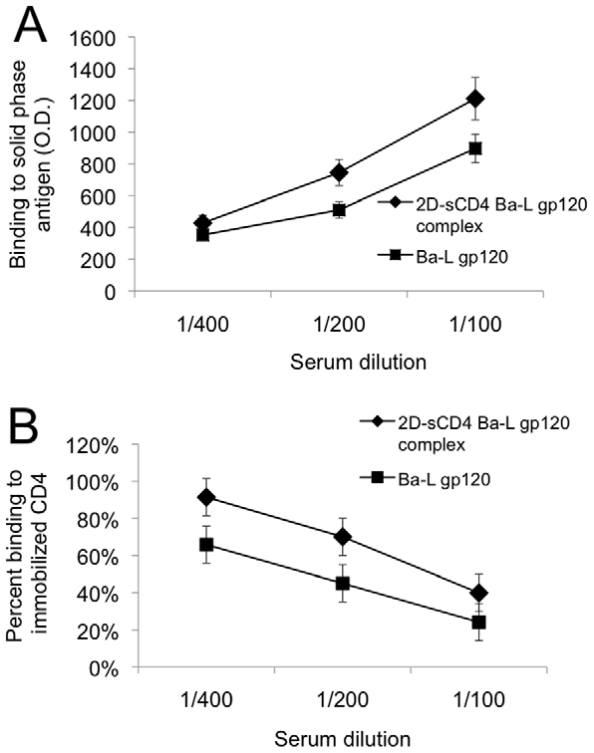
Biological effects of sera derived from mice immunized with CD4-complexed HIV-1 Env. A. Binding of pooled sera derived from mice immunized with HIV-1 Env in complex or not with soluble CD4 to solid phase gp120 (from isolate HIV-1 Ba-L). **B**. Inhibition of phosphatase-labeled Ba-L gp120 binding to solid-phase 2D-sCD4 by sera from mice immunized with HIV-1 Env in complex or not with soluble CD4. The phosphatase activity, measured by the rate of PNPP hydrolysis, is expressed as percent of the positive control (no serum added).

Sera from immunized mice were then tested in ELISA for their binding activity to HIV-1 gp120 and for their ability to block the binding of soluble gp120 to solid-phase CD4. Figures 3A and 3B show that sera obtained from mice immunized with sCD4-complexed Env displayed a higher binding activity for gp120 but a lower ability to interfere with sCD4-gp120 binding, compared to sera from mice immunized with uncomplexed Env. This result is compatible with the lack of exposure of the gp120-CD4 binding interface on the molecular complexes. Taken together, the results shown in Figures 2 and 3 suggest that immunization with sCD4-complexed Env elicited the production of antibodies lacking the ability to interfere with CD4-gp120 interaction in spite of their HIV-1-inhibitory activity.

### Characterization of hybridomas derived from mice immunized with NIH-3T3 cells expressing sCD4-complexed HIV-1 Env

Culture supernatants from hybridomas derived from mice immunized with NIH-3T3 cells expressing sCD4-complexed HIV-1 Env were tested for their IgG binding activity against different molecular targets. The hybridomas were negatively selected for production of IgG binding to uninfected NIH-3T3 cells and positively selected for production of IgG binding to either 2D-CD4, gp120 (Ba-L) or the 2D-CD4-gp120 (Ba-L) complex. A total of 25 hybridomas producing MAbs directed against either gp120 or 2D-CD4 were obtained (Table 1). Eight of these 25 MAbs displayed a higher binding activity against the CD4/gp120 complex than against the uncomplexed molecules. Surprisingly, however, unlike several MAbs described in the literature that are directed against CD4-induced epitopes in gp120 [11], [36], [37], all the MAbs derived from our mice with complex-enhanced binding activity were reactive with the CD4 moiety of the complex (Table 1).

**Table 1:** Characteristics of the IgG-secreting hybridomas obtained from immunization of Balb-c mice with native HIV-1 Env complexed with sCD4.

Well no.	Binding to CD4	Binding to gp120	Binding to CD4-gp120
A1	2.203	0.045	1.321
A2	2.342	0.035	1.221
A3	3.222	0.032	0.982
A9	0.040	2.121	2.001
A10	0.045	2.980	2.322
A11	0.034	1.342	1.032
A12	0.098	1.323	1.120
B4	0.043	0.430	0.462
B6	0.087	0.324	0.231
B6	0.087	0.342	0.231
B12	2.120	0.131	2.241
C1	1.320	0.011	1.232
C2	1.983	0.198	1.763
C3	1.348	0.022	1.134
C8*	1.221	0.045	1.430
C9*	1.234	0.023	1.998
C10*	1.432	0.042	1.743
C11*	1.565	0.079	2.211
C12*	0.092	1.211	1.243
D1	0.102	0.942	0.872
D2	0.023	1.234	1.356
D3	0.113	0.534	0.435
D10*	1.234	0.045	2.311
D11*	1.342	0.023	2.432
D12*	1.532	0.065	2.543

Binding activity to the indicated solid-phase antigens of IgG present in culture supernatants from hybridomas derived from spleen cells of mice immunized with NIH-3T3 cells infected with vaccinia recombinant vCB43, expressing Env from the R5 HIV-1 isolate Ba-L, and complexed with 2-domain sCD4. Binding activity was measured with culture supernatants diluted 1∶1 (see methods). Values are qualitative, since different microcultures contained different numbers of cells. Asterisks indicate supernatants where an increased binding to the 2D-CD4/gp120 (Ba-L) complex was observed as compared to the binding to each moiety separately.

### Characterization of MAb DB81

**Figure 4:**
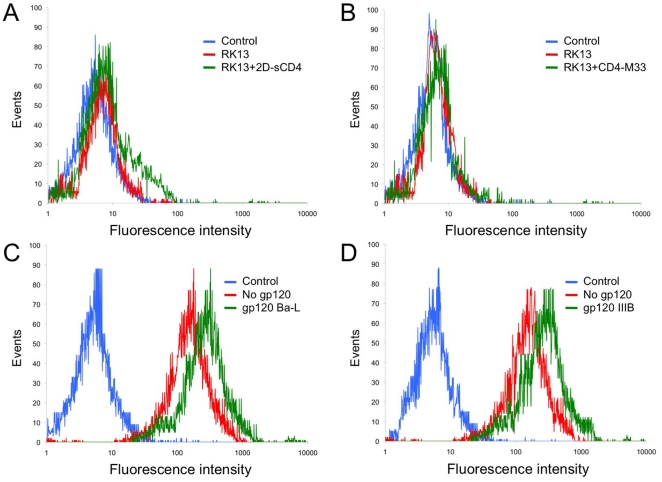
Membrane expression of the DB81 epitope. **A**. Cytofluorimetric analysis of MAb DB81 binding to RK13 cells expressing HIV-1 Ba-L and treated or not with soluble CD4. A FITC-conjugated, affinity purified polyclonal antibody to mouse IgG was used as a secondary reagent. The blue line shows background binding of the secondary antibody; the red line shows binding of MAb DB81 to untreated cells; the green line shows binding of MAb DB81 to cells pre-treated with 2D-CD4. **B.** Cytofluorimetric analysis of MAb DB81 binding to RK13 cells expressing HIV-1 Ba-L and treated or not with a CD4 miniprotein (CD4-M3). A FITC-conjugated, affinity purified polyclonal antibody to mouse IgG was used as a secondary reagent. The blue line shows background binding of the secondary antibody; the red line shows binding of MAb DB81 to untreated cells; the green line shows binding of MAb DB81 to cells pre-treated with CD4-M3. **C, D.** Cytofluorimetric analysis of MAb DB81 binding to PM-1 cells treated or not with HIV-1 gp120. A FITC-conjugated, affinity purified polyclonal antibody to mouse IgG was used as a secondary reagent. The blue line indicates background binding of the secondary antibody; the red histogram shows binding of MAb DB81 to untreated PM-1 cells; the green histogram shows binding to PM-1 cells pre-treated with recombinant gp120 Ba-L (**C**) or recombinant gp120 IIIB (**D**), respectively.

To investigate the epitope specificity of the anti-CD4 MAbs with complex-enhanced binding activity, a hybridoma that exhibited preferential recognition of the CD4-gp120 complex (C9, IgG1*k;* Table 1) was selected for further studies. The stable antibody-producing hybridoma was sub-cloned at 0.2 cells per well and a single clone (DB81) was selected and expanded. Flow cytometric analysis showed that MAb DB81 recognizes membrane-expressed CD4 on uninfected PM-1 cells, as well as 2D-2CD4 bound to oligomeric Ba-L gp160 expressed by a recombinant vaccinia virus in RK13 cells (Fig. 4A). In contrast, no binding was observed to the complex formed by the Ba-L Env bound to a CD4 miniprotein, CD4-M3 (Fig. 4B), which contains only the core residues of the gp120-binding region of CD4 but is fully capable of inducing CD4-like conformational changes in gp120. This result further supports the notion that the DB81 epitope maps exclusively to the CD4 molecule and does not involve gp120.

In agreement with the ELISA data, an increased binding activity of MAb DB81 was observed when uninfected PM-1 cells were treated with soluble gp120 derived from either isolate IIIB (Fig. 4C) or isolate Ba-L (Fig. 4D), further demonstrating that the DB81 epitope is not involved in the gp120-binding interface and is better exposed following interaction with gp120. No binding competition was observed with a panel of anti-CD4 MAbs, including MAb 55, OKT4, OKT4A, Leu3a, SIM-1 and SIM-4, as assessed by either competitive flow cytometry or ELISA assays (not shown). Likewise, binding of MAb DB81 to solid-phase human 2D-CD4 and 4D-CD4 was increased by pre-incubation of the molecule with recombinant gp120 from either the Ba-L or IIIB strain (not shown). These data demonstrate that MAb DB81 is directed against a previously unrecognized gp120-induced CD4 epitope.

### Reactivity of MAb DB81 with CD4 from nonhuman primates and preliminary mapping of the DB81 epitope

**Figure 5:**
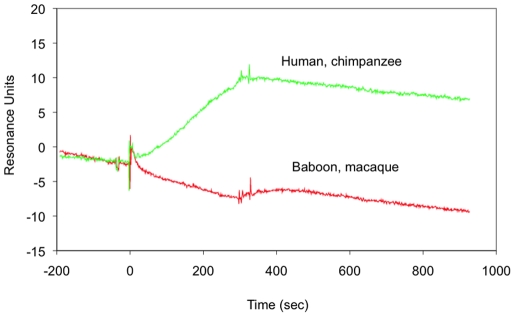
BIACORE® sensogram of interactions between MAb DB81 (bound to the chip) and peptides GTWTCTVLQNQKKVEFKID (human, shown as a green line) or peptide GTWTCTVSQDQKTVEFKID (baboon, shown as a red line). The peptides correspond to aa. 180-198 of membrane CD4 from Hominidae (e.g., *Homo* and *P. troglogites*) and Cercopithecidae (e.g., *Macaca*), respectively.

As a first step toward a precise delineation of the DB81 epitope, we tested the reactivity of MAb DB81 against peripheral blood T cells derived from several nonhuman primate species, whose CD4 presents distinct, albeit limited, amino acid sequence diversity. As indicated in Table 2, MAb DB81 was reactive with T cells derived from humans and chimpanzees (*Pan troglodytes*), but not derived from baboons (*Papio anubis*) or from three species of macaques (*Macaca mulatta, fascicularis* and *nemestrina*). Sequence alignment of the CD4 protein from the 6 species examined revealed a surface-exposed loop region (aa. 184-196) in the second Ig-like C2-type 1 domain containing two nonconservative amino acid substitutions that clearly segregated with MAb DB81 reactivity (Table 2). To evaluate the role of this region in MAb DB81 reactivity, we synthesized two 19mer peptides containing either the human/chimpanzee sequence (L187, K192) or the baboon/macaque sequence (S187, T192), and tested their reactivity with MAb DB81 in ELISA. As shown in Figure 5, surface plasmon resonance clearly demonstrated that MAb DB81 was reactive with the human CD4-derived peptide but failed to react with the baboon/macaque- CD4-derived peptide, strongly suggesting that this region contains the epitope recognized by the MAb. Analysis of the published tridimensional structures of human CD4 in the unliganded and gp120-bound state revealed small but significant conformational differences in the putative DB81 epitope (Figure S1).

**Table 2:** Sequence alignment of segment 126-204 from the second Ig-like domain of the CD4 molecule from different primate species.

Species	Sequence	DB81 epitope
*H. sapiens*	-TANSDTHLLQGQSLTLTLESPPGSSPSV**Q**CRSP**R**GKNIQGGKTLSVSQLE**L**QDSGTWTCTV**L**Q**N**QK**K**VEFKIDIVVLAF-	yes
*P. anubis*	-TANSDTHLLEGQSLTLTLESPPGTSPSV**K**CRSP**R**GKNIQGGRTL - - ----**-**-----**-**--NV**S**Q**D**QK**T**VEFKIDIVVLAF-	no
*M. nemestrina*	-TANSDTHLLEGQSLTLTLESPPGSSPSV**K**CRSP**G**GKNIQGGRTLSVPQLE**R**QDSGTWTCTV**S**Q**D**QK**T**VEFKIDIVVLAF-	no
*P. troglodytes*	-TANSDTHLLQGQSLTLTLESPPGSSPSV**Q**CRSP**R**GKNIQGGKTLSVSQLE**L**QDSGTWTCTV**L**Q**N**QK**K**VEFKIDIVVLAF-	yes
*M. mulatta*	-TANSDTHLLEGQSLTLTLESPPGSSPSV**K**CRSP**G**GKNIQGGRTISVPQLE**R**QDSGTWTCTV**S**Q**D**QK**T**VEFKIDIVVLAF-	no
*M. fascicularis*	-TANSDTHLLEGQSLTLTLESPPGSSPSV**K**CRSP**G**GKNIQGGRTLSVPQLE**R**QDSGTWTCTV**S**Q**D**QK**T**VEFKIDIVVLAF-	no
	-*********:*************:**** **** *******:******** ********** * ** ************-	

Sequences (amino-acids 126 to 204) were obtained from UniProt under the following accession numbers: *Homo sapiens* (P01730), *Papio anubis* (C1JYS8 and B1NC12), *Macaca nemestrina* (Q08340), *Pan troglodytes* (P16004), *Macaca mulatta* (P16003), *Macaca fascicularis* (P79185). The underlined sequences indicate the two synthetic peptides used for epitope mapping. Alignment was performed using the T-COFFEE software (version 1.41) and with the Nomad (Neighborhood Optimization for Multiple Alignment Discovery) program. Asterisks indicate identity; colons indicate homology. Positions with non-conservative substitutions between human and nonhuman primate sequences are in bold.

### Binding kinetics of MAb DB81 to CD4 or CD4-gp120 complexes

**Figure 6:**
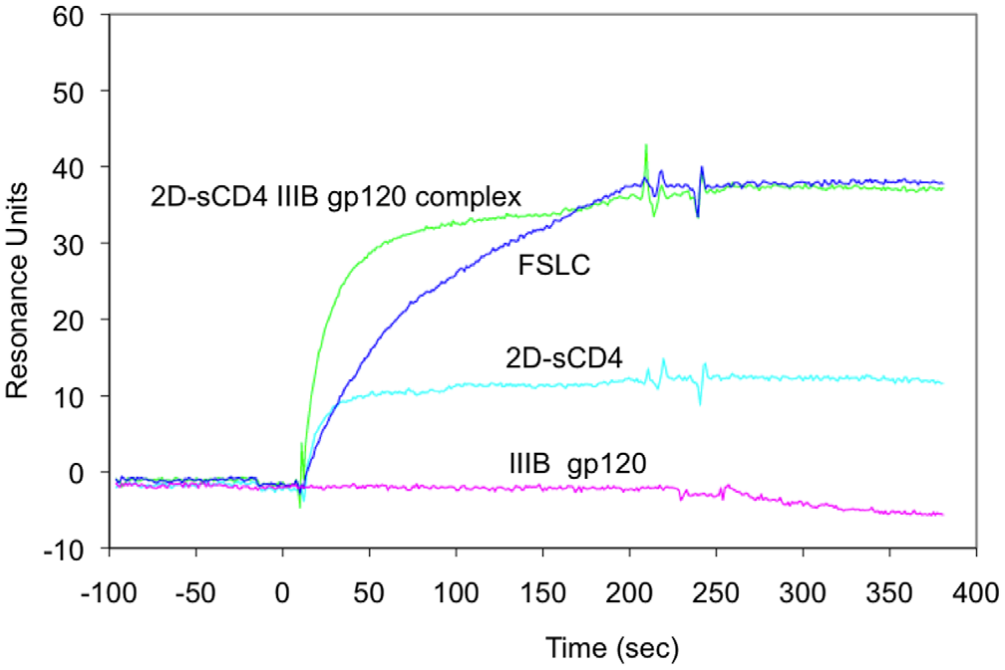
BIACORE® sensogram of interactions between MAb DB81 (bound to the chip) and 2D-CD4 (light blue), gp120 IIIB (violet), CD4-gp120 IIIB complex (green) or the FLSC chiimeric protein (dark blue).

By surface plasmon resonance, we measured the specificity and kinetic rates of binding and dissociation of MAb DB81 to either sCD4, gp120, the sCD4-gp120 complex, or a previously described chimeric single-chain molecule, designated FLSC, encompassing full-length HIV-1Ba-L gp120 and the D1D2 domains of CD4 joined by a 20-amino-acid linker. Figure 6 depicts the sensogram of these interactions, which were measured with MAb DB81 bound to the solid-phase chip. Binding of MAb DB81 to 2D-CD4 was clearly increased when CD4 was complexed with gp120 from isolate IIIB (45 versus 16 resonance units), confirming the results obtained by ELISA (Figure S2). When gp120 from isolate Ba-L was used, a lower increase was observed (not shown). The chimeric FLSC peptide was also recognized but showed a much slower slope of association with MAb DB81 compared to the CD4-gp120 complex. In contrast, no binding was measured with gp120 alone either from isolate IIIB (Figure 5) or from isolate Ba-L (not shown).

The kinetics of MAb DB81 binding were measured on either 2D-CD4 or the pre-formed CD4-gp120 complex. As shown in Table 3, MAb DB81 bound 2D-CD4 with an affinity of 8.6 nM, but the affinity increased approximately 800 times (9.6 pM) when 2D-CD4 was complexed with gp120 IIIB. From the binding kinetics, it is evident that the main parameter responsible for this change was not the association constant, which was quite high for both ligands (∼10^5^ M-1s-1), but rather the dissociation constant that was 2.7×10^-3^ s-1 for 2D-CD4 and 2.9×10^-6^ s-1 for the CD4-gp120 complex. Notably, the affinity of MAb DB81 for FSLC was roughly five times lower as compared to that for CD4, a results that mainly resulted from a remarkably low on-rate, ∼3 logs lower than for CD4 and the complex, suggesting that the accessibility of the DB81 epitope was impaired in this construct. In contrast, the off-rate was quantitatively close to that of the CD4-gp120 complex, a result compatible with the structure of FSLC, which is a mimetic of the CD4-gp120 complex.

**Table 3:** Affinities and binding kinetics of MAb DB81.

Analyte	K_d_ (×10^-9^ M)	K_on_ (×10^5^ M^-1^s^-1^)	K_off_ (×10^-3^s^-1^)
**CD4**	8.57	3.17	2.7
**CD4 + gp120_IIIB_**	0.0096	3.10	0.0029
**FLSC**	44.9	0.00231	0.01

Association (K_on_) and dissociation (K_off_) rate constants for purified MAb DB81 were measured by surface plasmon resonance. The K_d_ was calculated as K_off\_\K_on_.

### Effect of MAb DB81 on HIV-1 Env-mediated fusion and infection

**Figure 7:**
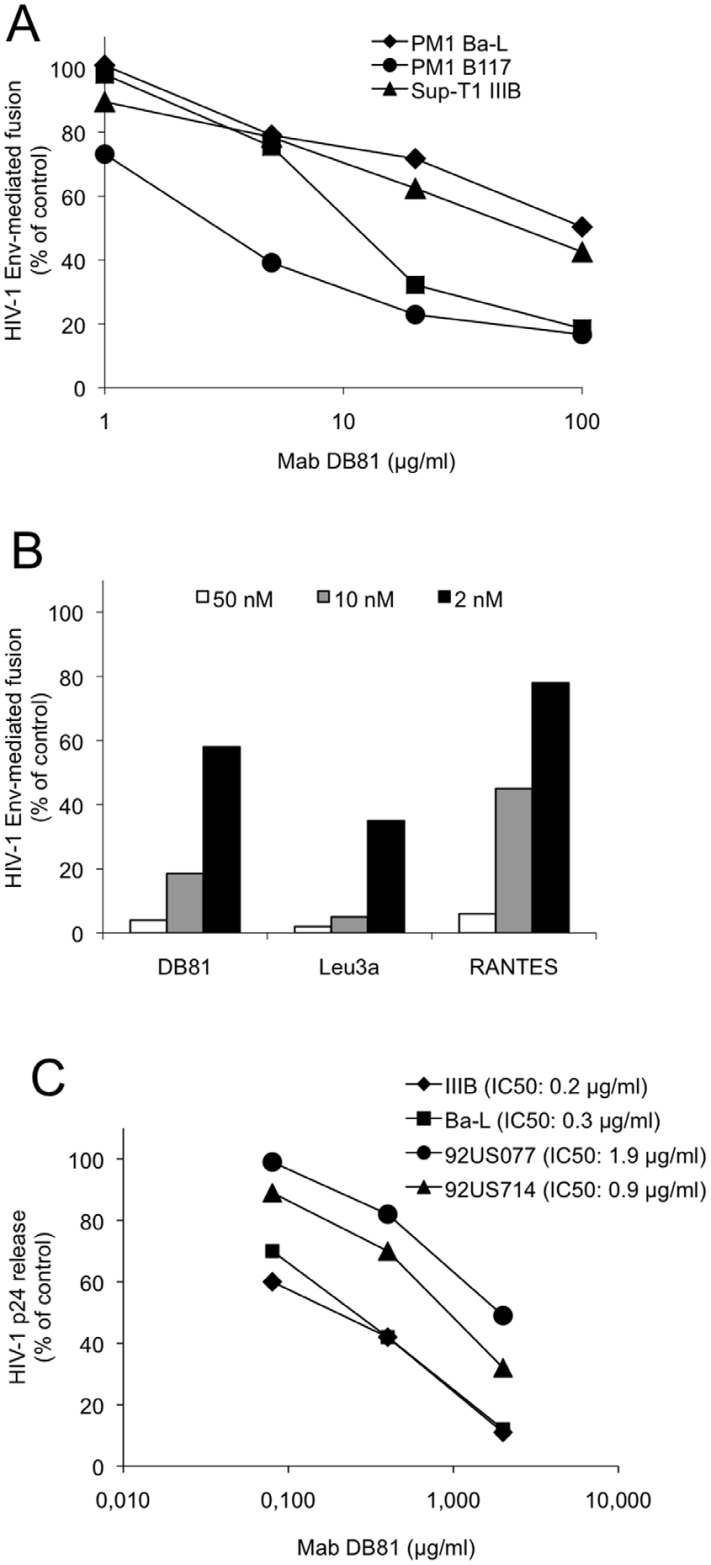
Inhibitory effect of MAb DB81 on HIV-1 Env-mediated fusion and infection. **A.** Effect of MAb DB81 on HIV-1 Env-mediated fusion. Chronically infected PM-1 cells were used as effector cells. NIH 3T3 cells expressing CD4 and the appropriate coreceptor were used as target cells. Fusion inhibition is expressed as percent of the fusion obtained with the untreated control (no antibody added). **B**. Comparison of the inhibitory potency of MAb DB81 with that of other HIV-1 inhibitors. Comparison of fusion inhibition by MAb DB81 with that obtained with equimolar amounts of another mAb to human CD4 (Leu3a) or the coreceptor inhibitor RANTES. PM-1 cells chronically infected with HIV-1 Ba-L were used as effectors. NIH 3T3 cells expressing the CCR5 coreceptor were used as targets. Results are expressed as percent of the fusion measured in the positive control (no inhibitors added). **C.** Effect of MAb DB81 on HIV-1 infection in primary human PBMC. PBMC from healthy blood donors were infected with the indicated HIV-1 strains in the presence or absence of MAb DB81. HIV-1 92US077 and 92US714 are two primary isolates derived directly from patient blood cells and minimally passaged *ex vivo* exclusively in primary cells. HIV-1 replication was measured after 4 days by p24 ELISA and expressed as percent of p24 measured in control untreated cultures. IC50 values were calculated using the PRISM software.

Next, we examined the ability of MAb DB81 to block fusion mediated by a panel of HIV-1 Envs derived from both laboratory adapted and primary isolates. As shown in Figure 7A, MAb DB81 efficiently inhibited fusion mediated by the Env of three different HIV-1 isolates with diverse coreceptor-usage phenotype, Ba-L (R5), B117 (R5X4), IIIB (X4) and 6195 (R5X4). In these assays, MAb DB81 showed a potency similar to that of Leu3A, a reference anti-CD4 MAb directed against the gp120-binding site, and RANTES, a CCR5 ligand (Fig. 7B). Taken together, these results indicate that the DB81 epitope is accessible on the native oligomeric gp120, and is conserved in CD4-Env complexes formed by HIV-1 strains with different coreceptors-usage phenotype.

We also tested the ability of MAb DB81 to inhibit acute infection by different laboratory-adapted and primary HIV-1 isolates in primary PBMC cultures. PHA-activated PBMC were acutely infected with two laboratory-adapted isolates, IIIB (X4) and Ba-L (R5), and two primary isolates, minimally passaged *ex vivo,* 91US714 (R5) and 92US077 (R5X4). MAb DB81 was used to pre-treat the cells before infection and then maintained at the same concentration throughout the experiment. HIV-1 inhibition was evaluated as reduction of extracellular p24 Gag antigen production. The results, presented as half maximal inhibitory concentrations (IC50), demonstrate a potent inhibitory activity of MAb DB81 irrespective of the viral isolate tested (Fig. 7C).

### Effects of MAb DB81 on antigen-induced T-cell proliferation and complement fixation

**Figure 8:**
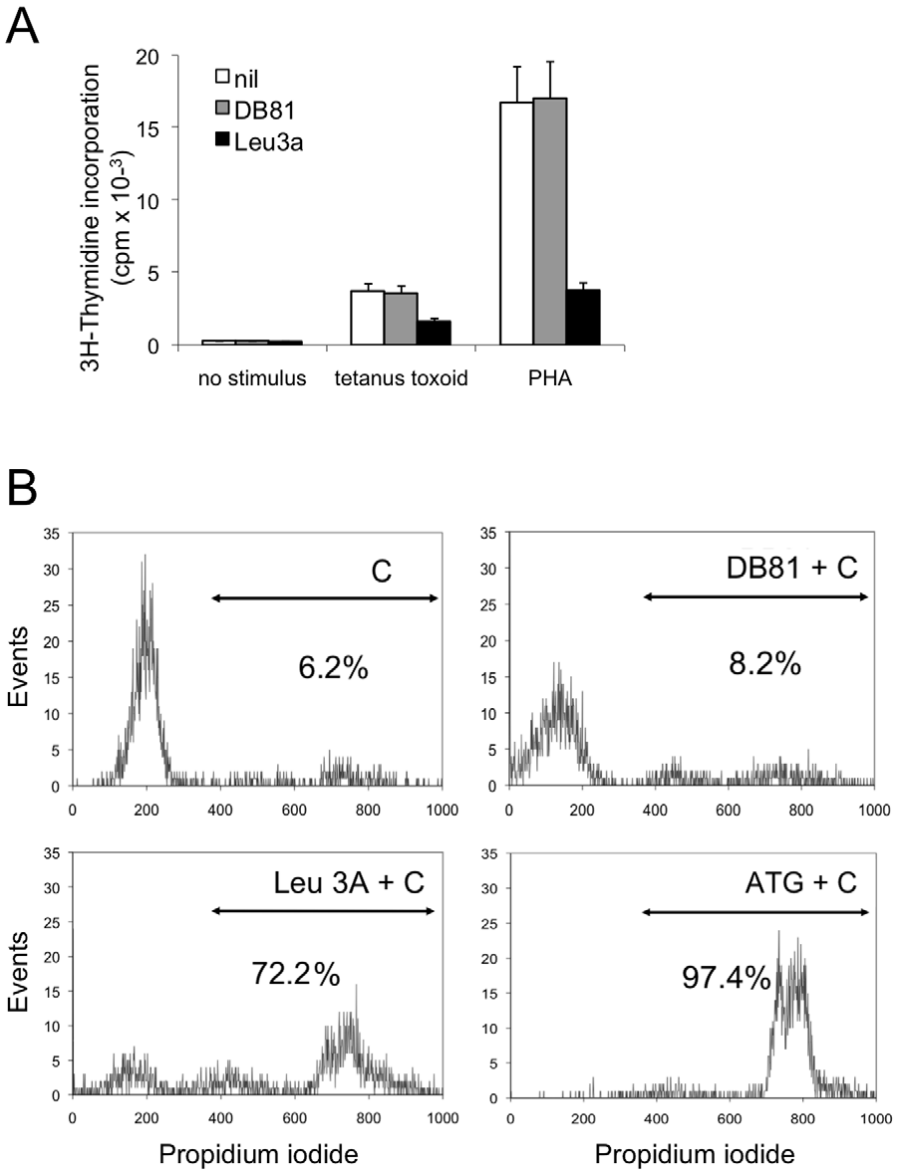
Immunosuppressive and complement-fixing effects of MAb DB81. **A.** Inhibition of PBMC proliferation by MAb DB81. PBMC from 4 healthy blood donors were incubated with the indicated stimuli in a 5-day proliferation assay. Either no inhibitors (“nil”) or the indicated mAbs (3 µg/ml) were added at the beginning of the culture time. Proliferation was measured by incorporation of tritiated thymidine in triplicate wells. The values indicate the mean ± standard error of the mean. **B.** Complement fixation by MAb DB81. Flow cytometry analysis of PM-1 cells treated with complement only (C, top left) or with C plus thymoglobulin (polyclonal rabbit antibodies to human thymocyte antigens, ATG, bottom right), C plus MAb Leu-3a (bottom left) or C plus DB81 (top right). Cells were stained with propidium iodide as a marker for cell death. Numbers in panels indicate the percent of stained (complement lysed, dead) cells, which fell within the double arrow markers in the different experimental settings.

Since a major concern with the potential clinical use of antibodies directed against T cell-expressed antigens is the risk of inducing immunosuppression, we evaluated the effects of MAb DB81 on nominal antigen-induced T-cell proliferation. As shown in Figure 8A, MAb DB81 did not exert inhibitory effects on the proliferation of primary human PBMC stimulated with a nominal antigen (tetanus toxoid) or with a polyclonal activator (PHA), while the isotype-matched anti-CD4 MAb Leu3a displayed marked suppressive effects. MAb DB81 was also poorly efficient in inducing complement fixation and cytolysis, as measured by propidium iodide incorporation following exposure of MAb-treated cells to rabbit complement; again, the isotype matched MAb Leu3a displayed a markedly higher activity (Fig. 8B).

### Sequence analysis of MAb DB81 genes

Both the light and heavy chains of the MAb DB81 gene were sequenced. Analysis of the light chains by the international ImMunoGeneTics information system®(IMGT) [38] assigned the V-gene to the IgK V6-17*01 gene family and the J-gene to the IgK J2*01 gene family, with levels of identity of 97.8 and 88.2%, respectively. The VK complementary determining region (CDR) 1, CDR2 and CDR3 lengths were 6, 3 and 9 amino acids, respectively; the amino acid sequence in the junction encompassing framework (FR) 3 and CDR3 was CQQHYSTPPTF. The complete sequence of the light chain of MAb DB81 is available in the EMBL database under accession number CAQ16368. Analysis of the FR and CDR delimitations of the MAb DB81 light chain is shown in Table 4.

**Table 4:** Framework (FR) and Complementary Determining Region (CDR) delimitations of the light chain of MAb DB81: DB81 VK.

<--	---	---	---	---	---	---	---	---	---	---		FR1	-	IMGT
1				5					10					15
D	I	V	M	T	Q	S	H	K	F	M	S	T	S	V
gac	atc	gtg	atg	acc	cag	tct	cac	aaa	ttc	atg	tcc	aca	tca	gta

---	---	---	---	---	---	---	---	---	---	-->	___	___	___	___
				20					25					30
G	D	R	V	S	I	T	C	K	A	S	Q	D	V	S
gga	gac	agg	gtc	agc	atc	acc	tgc	aag	gcc	agt	cag	gat	cag	agt

___	CDR1	-	IMGT	___	___	___	___	<--	---	---	---	---	---	---
				35					40					45
T	T							V	A	W	Y	Q	Q	K
act	act	...	...	...	...	...	...	gtg	gcc	tgg	tat	cag	cag	aaa

FR2	-	IMGT	---	---	---	---	---	---	-->	___	___	___	___	CDR2
				50					55					60
P	G	Q	S	P	K	L	L	I	Y	S	A	S		
cca	ggg	caa	tct	cct	aaa	cta	ctg	att	tat	tcg	gca	tcc	...	...

-	IMGT	___	___	___	<--	---	---	---	---	---	---	---	---	---
				65					70					75
					Y	R	Y	T	G	V	P		D	R
...	...	...	...	...	tac	cgg	tac	act	gga	gtc	cct	...	gat	cgc

---	---	---	---	---	---	---	---	FR3	-	IMGT	---	---	---	---
				80					85					90
F	T	G	S	G			S	G	T	D	F	T	F	T
ttc	act	ggc	agt	gga	...	...	tct	ggg	acg	gat	ttc	act	ttc	acc

---	---	---	---	---	---	---	---	---	---	---	---	---	-->	___
				95					100				104	
I	S	S	V	Q	A	E	D	L	A	V	Y	Y	C	Q
atc	agc	agt	gtg	cag	gct	gaa	gac	ctg	gca	gtt	tat	tac	tgt	cag

___	___	CDR3	-	IMGT	___	___	___	___	___	___	___	___	___	___
														
Q	H	Y	S	T	P	P	T	F	G	G	G	T	K	L
caa	cat	tat	agt	act	cct	ccc	acg	ttc	gga	ggg	ggc	acc	aag	ctg

___	___													
														
E	I	R												
aa	ata	cgg												

Analysis of the heavy chain of MAb DB81 assigned the V-gene to the IgH V1-39*01 family and the J-gene to the IgH J3*01 family, with identity levels of 91.7 and 82.3%, respectively. The D-gene was assigned to the IgH D2-2*01 family, in reading frame 3. The VH CDR1, CDR2 and CDR3 lengths were 8, 8 and 12 amino acids, respectively; the amino acid sequence in the junction encompassing FR3 and CDR3 was CAREGDYGHPFAYW. The complete sequence of the heavy chain of MAb DB81 is available in the EMBL database under accession number CAQ16367. Analysis of the FR and CDR delimitations of the MAb DB81 heavy chain is shown in Table 5.

**Table 5:** Framework (FR) and Complementary Determining Region (CDR) delimitations of the heavy chain of MAb DB81: DB81 VH.

<--	---	---	---	---	---	---	---	---	---	---	---	FR1	-	IMGT
1				5					10					15
E	V	Q	L	Q	Q	S	G	P		E	L	V	K	P
gaa	gta	cag	ctg	caa	cag	tct	gga	cct	...	gaa	ctg	gtg	aag	cct

---	---	---	---	---	---	---	---	---	---	-->	___	___	___	___
				20					25					30
G	A	S	V	K	I	S	C	K	A	S	G	Y	S	F
ggg	gct	tca	gtg	aag	ata	tcc	tgc	aag	gct	tct	ggt	tat	tca	ttc

___	CDR1	-	IMGT	___	___	___	___	<--	---	---	---	---	---	---
				35					40					45
T	D	Y	T					M	L	W	V	K	Q	S
act	gac	tac	acc	...	...	...	...	atg	ctc	tgg	gtg	aag	cag	agc

FR2	-	IMGT	---	---	---	---	---	---	-->	___	___	___	___	CDR2
				50					55					60
H	G	K	S	L	E	W	I	G	N	I	N	P	Y	Y
cat	gga	aag	agc	ctt	gag	tgg	att	gga	aat	att	aat	cct	tac	tat

-	IMGT	___	___	___	<--	---	---	---	---	---	---	---	---	---
				65					70					75
D	T	T			T	Y	N	L	N	F	K	…	G	K
gat	act	act	...	...	acc	tac	aat	ctg	aac	ttc	aag	...	ggc	aag

---	---	---	---	---	---	---	---	FR3	-	IMGT	---	---	---	---
				80					85					90
A	T	M	T	V	D	K	S	S	S	T	A	Y	M	Q
gcc	aca	atg	act	gta	gac	aaa	tct	tcc	agc	aca	gcc	tac	atg	cag

---	---	---	---	---	---	---	---	---	---	---	---	---	-->	___
				95					100				104	
L	N	S	L	T	S	E	D	S	A	V	Y	Y	C	A
ctc	aat	agt	ctg	aca	tct	gag	gac	tct	gca	gtc	tac	tac	tgt	gca

___	___	CDR3	-	IMGT	___	___	___	___	___	___	___	___	___	___
				110					115					120
R	E	G	D	Y	G	H	P	F	A	Y	W	G	Q	G
aga	gag	ggg	gac	tat	ggt	cac	cct	ttt	gct	tac	tgg	ggc	caa	ggg

___	___	___	___	___	___	___								
				125										
T	L	V	T	V	S	S								
act	ctg	gtc	act	gtc	tcg	agc								

## Discussion

In this study, we used an unconventional approach to immunize mice with the native, trimeric form of the HIV-1 Env complexed with recombinant soluble 2D-CD4, thus presenting the activated, fusion-competent conformation of Env. We succeeded in the elicitation of antibodies that recognized, in addition to constitutively expressed CD4-specific and Env-specific epitopes, a repertoire of complex-enhanced epitopes whose expression was functionally related to cell-fusion inhibition. A panel of MAbs was generated from Env-CD4 complex-immunized mice, which provided a useful tool for a more precise characterization of the specificity of the antibody response elicited by this unconventional immunization method. To our surprise, the MAbs that reacted with complex-enhanced epitopes were not directed against the gp120 moiety, as previously described for MAbs against CD4-induced epitopes [11], [36], [37] but rather to the CD4 moiety. Although we cannot exclude, in principle, the possibility that these MAbs recognize ‘bridging’ epitopes encompassing both CD4 and gp120, this hypothesis seems unlikely considering the complete lack of reactivity against the isolated gp120 moiety as well as against gp120 in its CD4-bound conformation induced by interaction with a CD4 miniprotein. Importantly, most of the elicited antibodies displayed HIV-1-neutralizing activity. Thus, our experimental system seems to have favored the elicitation of antibodies directed against CD4 epitopes that are conformationally modified upon binding to gp120, a feature that was never hitherto described for CD4. In fact, as we recently reported [39], comparison of the crystal structures of unbound and gp120-bound human CD4 reveals widely overlapping tridimensional conformations, without major alterations induced upon ligation. However, when C-α atom B-factors were considered as a measure of local backbone mobility, the second CD4 domain (D2) displayed large variations throughout its structure, whereas the first domain (D1) did not display any significant variation with the exception of the region that directly contacts gp120 [39]. This result suggests that the local flexibility of the D2 domain is significantly reduced upon binding to gp120, whilst the D1 domain remains virtually unaltered in its local mobility. This altered local mobility of D2 may influence the immunogenicity of CD4 by specifically exposing or masking specific residues. Moreover, it has to be emphasized that the existing crystal structures of the gp120-CD4 molecular complex were obtained using drastically truncated and fully deglycosylated gp120 core molecules, which might lack specific sites involved in the interaction with CD4. Thus, it is possible that CD4 undergoes more substantial conformational changes than it is currently appreciated after binding to the full-length, native gp120 molecule, which may have been missed due to the limitations of the existing structural models of gp120. The lack of previous reports of “gp120-induced” epitopes of CD4 may be a consequence of the primary focus of most investigators on gp120-specific epitopes or of the specific antigenic make-up of our immunogens, in which a soluble form of CD4 was complexed with the native, fully glycosylated, trimeric Env spike expressed on the surface of mammalian cells. In principle, immunogens expressing complex-specific epitopes on properly folded, membrane gp120 trimers are advantageous as compared to complexes formed with recombinant soluble molecules [40], [41]. In fact, the latter expose a large array of epitopes from the “silent” face of gp120, which remain embedded inside the core of the trimer in the native Env spike, and therefore are inaccessible to antibodies [2], [42]. For these reasons, our approach may prove effective in eliciting antibodies against conserved neutralization epitopes of potential relevance to a protective anti-HIV-1 vaccine.

Despite some potential advantages, several limitations have to be considered in the perspective of developing an effective vaccine based on native HIV-1 Env-CD4 complexes. First, immunized mice developed significant levels of anti-human CD4 antibodies. Although in humans the induction of anti-self antibodies should be restricted, this remains a concern for the risk of eliciting autoimmune reactions. Moreover, the need to employ fixed cells transduced with a recombinant vector for the expression of the HIV-1 Env in the fully native trimeric conformation poses additional safety concerns. Specifically, after repeated immunizations, we observed that tolerance to NIH 3T3 murine cells was partially broken, and individual mice started to produce antibodies to cellular antigens (not shown). Furthermore, the process of validation of our immunogens before injection revealed that the preparation of the vaccine was rather difficult to standardize, and roughly one third of the batches that we prepared had to be discarded before injection because they did not meet the quality criteria that we had established. This may be due to several reasons, including batch-to-batch differences in the expression of Env by vaccinia-infected cells, internalization of Env-CD4 complexes before fixation, or subtle changes in the fixation procedure itself. Nevertheless, our results establish the proof-of-principle that immunization with cell-surface-expressed native HIV-1 Env-CD4 complexes may be effective in eliciting the production of broadly neutralizing antibodies against HIV-1 Env and/or CD4, opening the way for further refinement of this vaccine approach.

One of the MAbs that we obtained from mice immunized with CD4-complexed HIV-1 Env (DB81) was thoroughly characterized. The epitope recognized by MAb DB81 was mapped to the outermost two domains of CD4 and more specifically to the vicinity of a 13-aa. loop in domain 2 to which none of the anti-CD4 MAbs so far reported seems to bind. In fact, MAb DB81 did not compete for binding with any other CD4-specific antibody tested. Of note, in contrast to previously reported monoclonal antibodies raised from mice injected with soluble CD4-gp120 complexes [43], MAb DB81 was reactive both with the solid-phase and with the membrane-bound molecule By plasmon resonance analysis, the binding affinity of DB81 to the CD4-gp120 complex was almost one order of magnitude higher than binding to the CD4 moiety alone. Moreover, the DB81 epitope was expressed on the FLSC peptide, a chimeric construct designed to express in a stable form the set of conformational epitopes that result from the post-binding reshaping of gp120 and CD4 [28]. Interestingly, an anti-CD4 antibody which recognizes a CD4 D2 epitope in proximity of the D1 CD4 domain (ibalizumab) [44] was recently tested in phase II clinical trials, in the form of humanized (IgG4) derivative, and appeared to be a promising tool to block HIV-1 infection without inducing any immunologically relevant side-effect either *in vitro* or *in vivo* [20], [45]. Of note, interference with conformational changes taking place at the post-binding level was indicated as the likely mechanism of action also for this antibody [46]. However, at variance with DB81, ibalizumab does not seem to preferentially bind to CD4 complexed to gp120 [46].

Of importance in the perspective of a potential clinical use, DB81 showed only a modest complement-fixation capability and no interference with antigen-induced T-lymphocyte proliferation *in vitro*. This limited immunosuppressive activity may be explained by the relatively modest CD4-binding activity in the absence of HIV-1 Env, or with the peculiar signaling that may be initiated *via* binding to the DB81 epitope [47]. Notably, MAb DB81 induced virtually no down-regulation of membrane CD4 on T lymphocytes (not shown), unlike immunosuppressive anti-CD4 antibodies [48], [49]. Moreover, similarly to ibalizumab, the specificity of MAb DB81 for the D2 domain of CD4 is also compatible with lack of immune suppression, since CD4 interaction with major histocompatibility complex class II (MHC-II) is mediated by the D1 domain [50]. These characteristics make DB81, or a derivative thereof, a promising entry inhibitor that may be potentially useful either for passive immunotherapy of HIV-1-infected individuals, or for early post-exposure prevention of HIV-1 infection.

In conclusion, the present study supports the concept that a vaccine based on native HIV-1 Env-CD4 molecular complexes may elicit antibodies against partially cryptic neutralization epitopes on both CD4 and gp120, which may have protective effects. Moreover, a specific antibody generated with this approach may be considered for the development of novel entry inhibitors for the treatment or prevention of HIV-1 infection.

## Supporting Information

Figure S1 Local conformation of the putative DB81 epitope in the unliganded (green; accession code: 1WIP/MMDB 6042) and gp120-bound (purple; accession code: 1G9N/MMDB 14984) tridimensional structures of human CD4. The main chains are depicted in the cartoon representation with arrows denoting beta-strands; the insert shows the same region with the main chains depicted as lines and the side chains as sticks. Small but significant differences are seen in the positioning and length of the beta-strands, as well as in the spatial orientation of the lateral chains, which can justify the preferential reactivity of MAb DB81 with the gp120-liganded form of CD4. In baboon and macaque CD4, residues Leu162, Asn165 and Lys167 are substituted by Ser, Asp and Thr, respectively.
(TIF)

Figure S2 Binding of MAb DB81 to solid phase CD4 or CD4-gp120 complexes. Binding of MAb DB81 to solid phase 2D-sCD4, either alone or in equimolar complex with different recombinant gp120 (from isolates Ba-L or IIIB) was measured by ELISA.
(TIF)
