# Supplementary figures and images for: Broad-Spectrum Inhibition of HIV-1 by a Monoclonal Antibody Directed against a gp120-Induced Epitope of CD4
Source: PLoS One. 2011 Jul 19;6(7):e22081. doi: 10.1371/journal.pone.0022081 (PMC3139607; doi:10.1371/journal.pone.0022081)

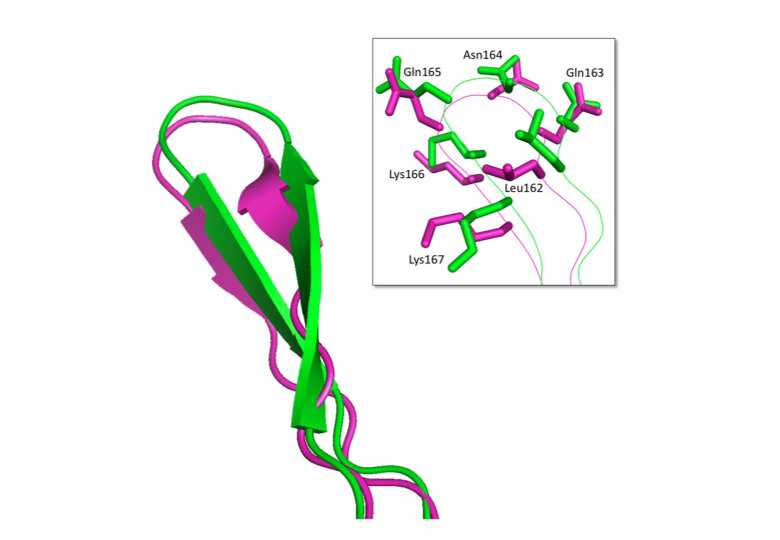

Supplement: Figure S1 — Local conformation of the putative DB81 epitope in the unliganded (green; accession code: 1WIP/MMDB 6042) and gp120-bound (purple; accession code: 1G9N/MMDB 14984) tridimensional structures of human CD4. The main chains are depicted in the cartoon representation with arrows denoting beta-strands; the insert shows the same region with the main chains depicted as lines and the side chains as sticks. Small but significant differences are seen in the positioning and length of the beta-strands, as well as in the spatial orientation of the lateral chains, which can justify the preferential reactivity of MAb DB81 with the gp120-liganded form of CD4. In baboon and macaque CD4, residues Leu162, Asn165 and Lys167 are substituted by Ser, Asp and Thr, respectively. (TIF) [file pone.0022081.s001.tif]

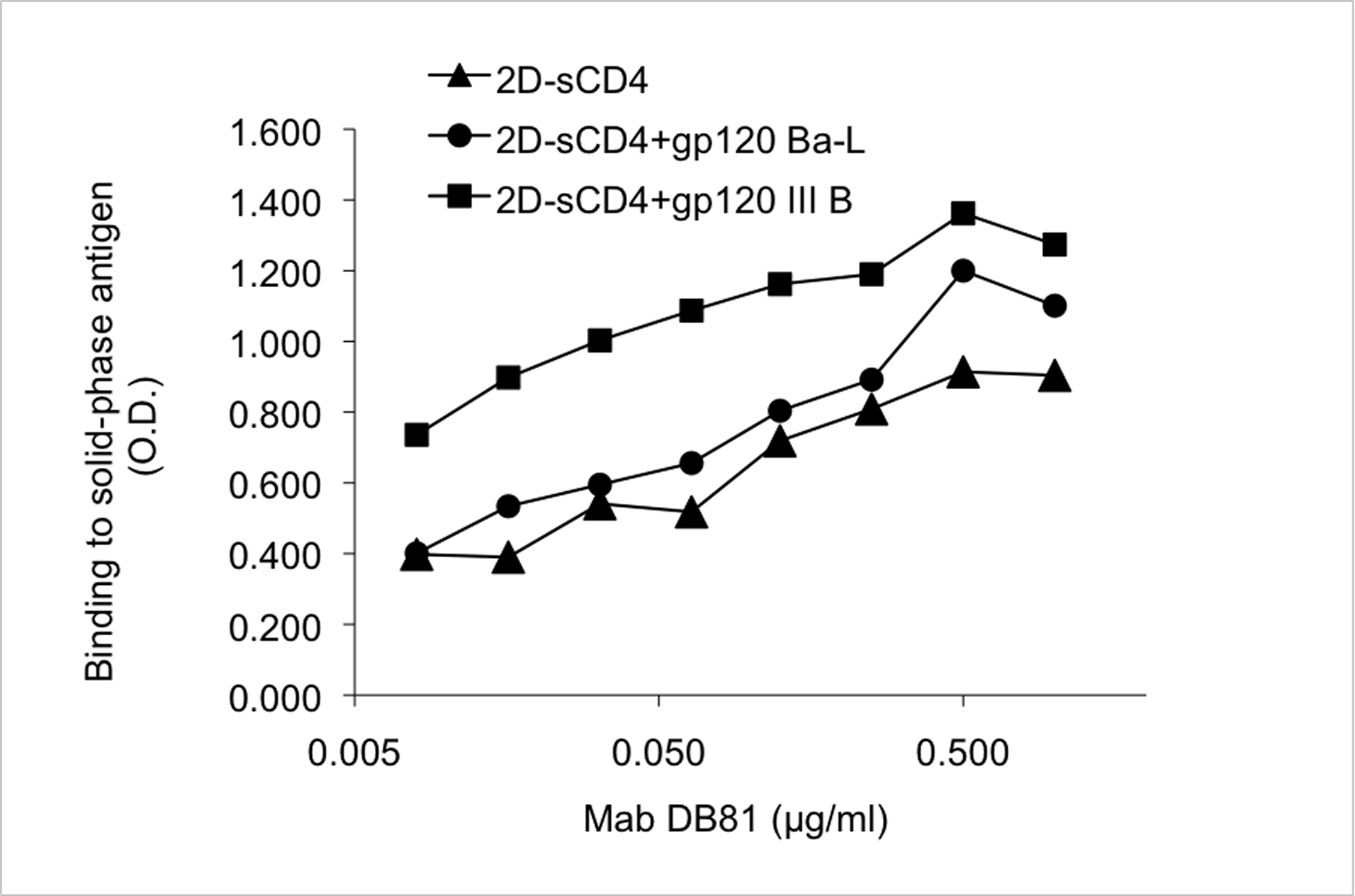

Supplement: Figure S2 — Binding of MAb DB81 to solid phase CD4 or CD4-gp120 complexes. Binding of MAb DB81 to solid phase 2D-sCD4, either alone or in equimolar complex with different recombinant gp120 (from isolates Ba-L or IIIB) was measured by ELISA. (TIF) [file pone.0022081.s002.tif]
